# On the Nature and Cause of the Buffy Coat of the Blood

**Published:** 1831-09

**Authors:** J. R. Taylor


THE LONDON

### Medical and Physical Journal.

391, VOL. LXVII.] SEPTEMBER 1831. [63, *New Series.*

"For many fortunate discoveries in medicine, and for the detection of numerous errors, the world is indebted to the rapid circulation of Monthly Journals; and there never existed any work to which the Faculty, in Europe and America, were under deeper obligations than to the 'Medical and Physical Journal of London,' now forming a long but invaluable series."—RUSH.

### ORIGINAL PAPERS, AND CASES,

### OBTAINED FROM PUBLIC INSTITUTIONS AND OTHER AUTHENTIC SOURCES.

### BUFFY COAT OF THE BLOOD.

### *On the Nature and Cause of the Buffy Coat of the Blood.*

By J. R. Taylor, Esq.

The nature and cause of the buffy coat so frequently found in the blood, under a great variety of circumstances, have always appeared subjects of some importance, and many physiologists, whose names alone carried sufficient weight to give value to any hypothesis emanating from them, have made these points the objects of their researches. There are, perhaps, few investigations that have produced a greater discrepancy of opinions, or theories more diametrically opposed to each other: all, however, seem to have failed in giving a satisfactory explanation, and the field is yet open to the scientific inquirer. We have theories to prove that the buff depends on stagnation of blood; that it results from increased velocity of circulation; others, again, attribute the phenomenon to unnatural fluidity; to these may be added many others, equally contradictory, and unfortunately inconclusive. The view I have taken is probably but one more added to the futile attempts already made; and if I have not been deterred by those which have preceded me, it is from no overweening confidence in my own powers; but if there be wisdom in many counsellors, truth may at last be elicited from many conflicting opinions, since, in science, we generally find that repeated failures, however disheartening at the time, eventually pave the way to success.

I have been led to believe that the blood becomes buffy from the addition of a matter to it, which is absorbed either by the veins themselves or the lymphatics communicating with them. This solution of the problem has appeared to me to explain many of the phenomena of disease observed in conjunction with this appearance of the blood, more satisfactorily than other theories to which I have alluded. It will be for the reader to compare the facts, and receive or reject the conclusions I have drawn, and which I shall now detail as concisely as possible.

The buffy coat I would consider, therefore, as something taken up from the body, and added to the circulating mass; and, further, that it becomes converted into arterial blood after having undergone the same processes in the heart and lungs as the matter conveyed into the circulation by the thoracic duct. Hence it is that arterial blood is very rarely buffy, and when this appearance is observed, we may conclude that the matter forming the buffy coat has been absorbed in too great a quantity to be converted into blood by passing through the heart and lungs.

The characteristic blueish-streaked appearance of buffy blood, when first drawn, gives rise to the idea of a whitish glutinous fluid having been added to the mass, but not thoroughly mixed with it. If this fluid be, as I suppose, absorbed matter, we can readily account for its separation, in the form of the buffy coat, from the want of that intimate admixture with the rest of the blood, and those chemical changes which take place in it before it enters the arteries. Indeed, considerable analogy may be traced between the buffy coat and the contents of the thoracic duct, both as regards its component parts and final conversion into arterial blood.

There is nothing new in supposing the buffy coat to be an addition to the venous blood, and not a part of its fibrin, from which the red particles have subsided during coagulation. Dr. Scudamore and Dr. Gregory support this opinion. Dr. Scudamore supposes that, in buffy blood, the fibrin is retained which ought to be distributed to the muscular fibre, and that its separation in the form of the buffy coat takes place from the red particles not being able to blend themselves with the whole quantity of the fibrin, as in ordinary coagulation.* Supposing, however, that no distribution of fibrin does take place, still this will not account for the separation of the red particles; for it is impossible that the venous blood can contain more fibrin than the arterial, unless some be added by absorption. Dr. Gregory, on the other hand, considers the buffy coat to be something retained

* Essay on the Blood, p. 117.

in the blood, from the deficient action of the organs engaged in the office of transpiration;* but this is rendered improbable by the fact that blood, drawn even when the patient is sinking under profuse perspirations, will be buffy. I agree, however, with these gentlemen in considering the buffy coat as something distinct from the venous blood, but I differ from them as to the mode of its production: absorption appears to me the most probable cause; for were the buffy coat composed of any thing which ought to have been secreted or thrown off, we should have evidences of it in the arterial as well as venous blood.

The constant occurrence of buffy blood whenever emaciation is going on rapidly, or to any extent, induced me to suspect that the matter forming the buffy coat was the result of the increased absorption going on in these cases; for the very circumstances which are known, by experiment, to increase absorption, also give rise to buffiness of the blood. Venous blood, for instance, presents the buffy coat when abstracted during the reaction following hemorrhage; and in cases of inflammation, also, the blood is very frequently found buffy at the second venesection, when it was not so at the first. Now, if it be true that bloodletting promotes absorption, these facts are strong arguments in favor of the theory I am endeavouring to explain. Buffy blood is also found to present itself in diseases attended with profuse local secretions, as consumption, diabetes, &c. In these cases the discharge is equivalent to loss of blood, and may therefore equally give rise to the absorption of the matter forming the buffy coat.

The possibility of the veins absorbing, or at any rate of conveying matters absorbed into the circulation, cannot be doubted. The experiments of MAGENDIE and Sir EVERARD HOME would tend to prove that the veins themselves absorb; while, on the other hand, Mr. ABERNETHY and several anatomists have traced absorbent vessels into veins, and numerous experiments have established the fact that they do communicate. In cases, then, where absorption is going on more rapidly, it seems but a natural deduction, from the anatomical structure of the parts, to suppose that the veins themselves absorb the matter forming the buffy coat, or that it is poured into them by the lymphatics. Why the absorbed matter, in these cases, should enter the veins, and not take the usual course to the heart, I am not prepared to say: perhaps the quantity may be too great for the absorbents to

* London Med. Gazette, vol. iii. p. 458.

carry, and therefore part passes into the veins; or it may even be possible that the lymphatics may not act at all, or but little in some cases, and the capillary veins do their duty.

Without at present inquiring into the cause of absorbed matter appearing in the veins, let us examine what would be its effects, and see how exactly they correspond with the circumstances accompanying buffiness of blood. Supposing then a matter, equal in quantity to the buffy coat, is constantly poured into the veins, the effect must be to increase the quantity of blood circulating in the body, unless some discharge exists at the same time equal to this supply. This is exactly what occurs when the blood is buffy: either we have evidences of too much blood being formed, as in many cases of acute inflammation, or the addition of the matter composing the buffy coat merely seems to prevent the arteries being too rapidly drained of their contents, as in cases of consumption, diabetes, hemorrhage, &c. We see now, therefore, why buffy blood is no indication of venesection, unless present in conjunction with other symptoms requiring that treatment.

It has long been remarked that patients bear the loss of blood better when suffering under inflammation, than other diseases of a different character. The quantity of blood in the body during inflammation appears to be increased, and any loss by venesection is more quickly renewed. Dr. Marshall Hall endeavours to explain this tolerance of loss of blood, by supposing inflammation to be "a sort of concentrated and permanent stimulus, exciting and maintaining the powers of the system."* No doubt a great deal depends upon an irregular distribution of the nervous power, but this is not all: the quantity of blood appears to me to be increased or kept up by the addition of the buffy matter, or patients could never recover from the profuse bleedings, so necessary in some cases of inflammation. Indeed, it was the opinion of Mr. Hunter that sizy blood contributed greatly to the support of the system under an increased local action; for he says, "women who are breeding, and are in perfect health, always have sizy blood, and this is most remarkably the case with all animals in similar situations: now, it would appear necessary for an animal, whenever put into a situation where greater powers are wanted, to have their powers increased. In pregnancy, there is a process going on which requires a greater exertion, or a greater quantity of power

* On the Morbid and Curative Effects of Loss of Blood, p. 203.

than usual, and therefore we have them produced."* Mr. Hunter, however, conceived that this greater quantity of power was effected by a real increase of the animal life, both of the solids and the fluids, and that the buffiness of the blood was the result of the increase of its vitality: but I think it will be considered more probable that the supply of power to the system, under an increased local action, arises rather from an increase of the quantity of the blood, than any increase of its vitality; for, in the first place, buffy blood is present when the animal life of the body is greatly diminished; and, secondly, there are cases where, if the supply afforded by the buffy coat were not constantly being added to the blood, the arteries would become emptied in a few days, from the excessive discharge occasioned by the increased local action. In consumption, for instance, where great quantities of pus and mucus are daily expectorated, and at the same time little or no blood formed by digestion, the patient would soon die of actual anemia, were it not for the supply of blood afforded by the absorption of the matter forming the buffy coat. In this, then, and all other increased local actions, natural or morbid, the buffy coat is present, forming the supply of power for the support of that action; and, as this supply is more requisite when the local action is high or attended with a great discharge, so is it in such cases more constantly found present.

This theory might further be strengthened by a comparison of the symptoms attending those cases in which a known absorption of matter has taken place, with the cases in which buffiness of the blood is most certainly and distinctly recognized. If we ask what are the symptoms of absorbed matter, are we not answered, great excitement, restlessness and a sense of oppression, a hard pulse, and a furred tongue? These, then, are precisely the most common symptoms in those states in which the blood is found greatly buffed. I cannot help thinking that questions of great interest, and even practical utility, arise from these considerations. Though this absorption of matter may be a salutary provision of nature to supply the powers of life when, from disease, there is great waste in the system, yet in other cases, where there is no such end to be accomplished, may not this peculiar action be brought on by vitiated functions, thus overloading and altering the character of the circulating fluid, and producing inflammatory disease? Do we not, in these cases, exactly imitate the process of nature already explained?

* Hunter on the Blood and Inflammation, p. 399.

we create a waste or a drain on the system; we bleed, weaken the vital powers, and diminish the quantity of vital fluid. We rarely, if ever, find a case of idiopathic inflammation, in which we cannot trace some preceding disordered function in the stomach, the alimentary canal, &c. Thus the diseased blood, if I may so term it, and the vitiated functions, may mutually act and react upon each other; some diseases may produce this state of blood; in others, a tendency to this peculiar character of the blood may bring on certain diseases, and any organ in which there may be a predisposition, from organic or accidental causes, will necessarily be the part attacked, and then we have deep-seated inflammation and disease of one or more organs, with or without that general inflammation which amounts to fever. What I wish to express is, that in some cases it may be the cause, at other times the result of disease, and that it may produce inflammation, but is by no means necessarily dependent upon it: in proof of this, we find that some diseases, of undoubted inflammatory character, exist without it,—enteritis, for example; while others, not generally considered inflammatory, as some kinds of dropsy, certain forms of fever, and apoplexy, are almost uniformly attended with it. If this be correct, as I believe, it will be evident that buffy blood may be, but is not certainly, a symptom of inflammation; its extraction may be, but is not necessarily, beneficial to the disease under which the patient is labouring; this depending, in a great measure, upon whether it be the cause or effect. How this may be ascertained, and how far it may be rendered of use in the treatment of disease, can only be determined by further research.

